# Evaluating the Role of High‐Dimensional Proxy Data in Confounding Adjustment in Multiple Sclerosis Research: A Case Study

**DOI:** 10.1002/pds.70112

**Published:** 2025-02-03

**Authors:** Mohammad Ehsanul Karim, Md. Belal Hossain, Huah Shin Ng, Feng Zhu, Hanna A. Frank, Helen Tremlett

**Affiliations:** ^1^ School of Population and Public Health University of British Columbia Vancouver British Columbia Canada; ^2^ Centre for Advancing Health Outcomes, University of British Columbia Vancouver British Columbia Canada; ^3^ Flinders Health and Medical Research Institute, College of Medicine and Public Health Flinders University Adelaide Australia; ^4^ SA Pharmacy SA Health Adelaide Australia; ^5^ Division of Neurology, Department of Medicine The Djavad Mowafaghian Centre for Brain Health, University of British Columbia Vancouver British Columbia Canada

**Keywords:** administrative health data, confounder adjustment, disease risk score, high‐dimensional data, multiple sclerosis, propensity score, residual confounding

## Abstract

**Purpose:**

Given the historical use of limited confounders in multiple sclerosis (MS) studies utilizing administrative health data, this brief report evaluates the impact of incorporating high‐dimensional proxy information on confounder adjustment in MS research. We have implemented high‐dimensional propensity score (hdPS) and high‐dimensional disease risk score (hdDRS) methods to assess changes in effect estimates for the association between disease‐modifying drugs (DMDs) and all‐cause mortality in an MS cohort from British Columbia (BC), Canada.

**Methods:**

We conducted a population‐based retrospective study using linked administrative databases from BC, including health insurance registries, demographics, physician visits, hospitalizations, prescriptions, and vital statistics. The cohort comprised 19 360 individuals with MS, followed from January 1, 1996, to December 31, 2017. DMD exposure was defined as at least 180 days of use for beta‐interferon or glatiramer acetate, or at least 90 days for other DMDs. The outcome was time to all‐cause mortality. We compared Cox proportional hazards models adjusting for investigator‐specified covariates with those incorporating additional empirical covariates using hdPS and hdDRS methods.

**Results:**

In the unadjusted analysis, DMD exposure was associated with a 69% lower risk of mortality (HR 0.31; 95% CI: 0.27–0.36). Adjusting for investigator‐specified covariates, the adjusted hazard ratio (aHR) was 0.76 (95% CI: 0.65–0.89). HdPS analyses showed a 20%–23% lower mortality risk (aHRs: 0.77 to 0.80), while hdDRS analyses indicated a 19%–21% reduction (aHRs: 0.79 to 0.81).

**Conclusions:**

Incorporating high‐dimensional proxy information resulted in minor variations in effect estimates compared to traditional covariate adjustment. These findings suggest that the impact of residual confounding in the question under consideration may be modest. Further research should explore additional data dimensions and replicate these findings across different datasets.

**Summary**

- The brief report demonstrates implementation of high‐dimensional proxy information from administrative health data in Multiple Sclerosis (MS) research.
- Both high‐dimensional propensity score (hdPS) and high‐dimensional disease risk score (hdDRS) methods showed consistent reductions in mortality risk estimates, supporting their utility in robust epidemiological analyses.
- The observed variations in effect estimates when using high‐dimensional methods were minor. However, this could suggest either that residual confounding in MS research may be less influential than previously assumed, or that the available proxy information was not sufficient to fully account for all unmeasured confounding in this setting.
- The findings validate the use of administrative health databases for long‐term outcome studies in MS, emphasizing their feasibility and cost‐effectiveness.
- The study highlights the need for further exploration of additional data dimensions and replication in diverse datasets to better understand and mitigate residual confounding in chronic disease research.

## 1 Purpose

Administrative health databases are arguably the most feasible and pragmatic data sources to analyze chronic diseases involving long‐term outcomes. For example, the average life expectancy for people living with multiple sclerosis (MS) is shorter by around 6 to 14 years [1, 2]. Specifically, a study in the province of British Columbia (BC), Canada showed a reduction in life expectancy of about 6 years among both males and females with MS than that of the age‐matched general population [3]. Conducting a clinical trial with mortality as the outcome in the MS population would clearly be prohibitively expensive due to the relatively long life expectancy of individuals with MS.

Disease‐modifying drugs (DMDs) are used to manage MS, but these DMDs are often associated with adverse effects. No randomized controlled trial has been conducted specifically to assess the association between MS DMDs and long‐term outcomes, such as all‐cause mortality, largely due to the logistical and financial challenges involved. Instead, administrative health data capturing information from virtually all residents (e.g., BC) offers us a practical and cost‐effective way of conducting analysis in a real‐world setting. Such data sources provide information to generate the following characteristics at the MS diagnosis: age, sex, neighborhood income, Charlson comorbidity index, and calendar year, all of which are necessary to consider in the analysis [4]. However, administrative data sources do not capture several lifestyle factors, for example, smoking behavior, alcohol consumption, and level of physical activity [5]. Although some MS‐specific details, such as MS disease duration, phenotype, or disability level, are available for a smaller group of individuals who visited one of the four major MS clinics in the region (and whose MS clinical and administrative data could be linked), they often are not adjusted for in the analysis as they are not entirely representative of those living with MS in the region [6]. Therefore, using this prior study on the MS population in BC, Canada as an example, residual confounding can be a cause for concern while analyzing health administrative data.

To reduce bias due to unmeasured confounding in health administrative data sources, the high dimensional propensity score framework has been proposed [7, 8]. Researchers identify useful proxy information from the same subjects using high‐dimensional health administrative data within this framework, and incorporate it into the analysis as additional covariates to reduce residual confounding. In this brief report, we apply this framework to incorporate high‐dimensional proxy information for confounder adjustment and investigate the association between MS DMDs and all‐cause mortality, comparing the results to analyses solely adjusted for investigator‐specified covariates. Rather than conducting a head‐to‐head methodological comparison—which has been extensively examined in prior simulation research [9, 10, 11, 12]—this brief report emphasizes the practical implementation of these methods and their impact on effect estimates within a case study.

## 2 Methods

### 2.1 Study Design and Data Sources

We used a population‐based observational retrospective study design. Briefly, the cohort was developed based on linked health administrative databases from January 1, 1996 to December 31, 2017, including provincial health insurance registries (which captured demographics such as sex, birthdates, residency status, and place of residency), physician visits and hospitalizations (which included diagnoses using ICD‐9/10 codes), prescription data (which captured medications dispensed at outpatient and community pharmacies, including dates, unique drug identification numbers, and number of days supplied), and vital statistics (which provided death dates) [5]. Further details have been described elsewhere [5].

### 2.2 Study Population

The study population includes people with MS identified using information from physician visits, hospitalizations, and prescriptions filled. We used a validated algorithm that requires ≥ 3 MS‐specific physician visits and/or hospitalizations with an ICD‐9/10 codes 340/G35, or one prescription filled for an MS DMD ever [5, 13].

The index date (representing the start of follow‐up) was the most recent of the first MS or related demyelinating disease diagnostic code or MS DMD prescription (Table S1) filled, the person's 18th birthday, or 1 January 1996. The study end date was the earlier of death, emigration from the province (measured as the date of cancelation of the mandatory provincial health insurance plan), or 31 December 2017.

### 2.3 Exposure

Exposure to any DMD (binary) was the variable of interest. We defined exposure to any DMD from prescription data as ≥ 180 days of cumulative use for beta‐interferon or glatiramer acetate, or ≥ 90 days of cumulative use for natalizumab, fingolimod, dimethyl fumarate, or teriflunomide, guided by prior studies [14, 15, 16, 17]. The duration of DMD exposure was measured using the days supplied. Gaps in DMD supply for the same DMD class of ≤ 30 days were allowed when calculating continuous exposure [18]. To avoid time‐varying analysis in favor of intuitive interpretation of the treatment effect estimate, we considered ever versus never exposed to any DMD in the 5 years post‐index date. Participants who initiated DMDs after the specified exposure window (5 years postindex date) were also censored at that time.

We have also performed a number of sensitivity analyses changing the exposure period to 3, 4, and 6 years. Later, we have compared these estimates with previous literature where analysis was conducted using a time‐varying exposure [5].

### 2.4 Outcome

The outcome of interest was the time from the index date to the date of all‐cause mortality. Mortality data were extracted from the vital statistics deaths database. Censoring was applied to account for participants who were lost to follow‐up, emigrated from the province (as indicated by cancelation of provincial health insurance), or reached the study end date (December 31, 2017) without experiencing the outcome of interest (all‐cause mortality).

### 2.5 Investigator‐Specified Covariates

All covariates were defined in a one‐year window prior to the index date [19]. We considered the following investigator‐specified covariates based on the literature [5, 20, 21]: age (continuous), sex (male/female), neighborhood income quintiles, comorbidity status (0, 1, 2, ≥ 3 comorbidities present), and calendar year (1996–1999, 2000–2005, 2006–2011, or 2012–2017). Comorbidity status was assessed using a modified weighted Charlson Comorbidity Index [22], where hemiplegia/paraplegia was excluded from the index to avoid misclassifying MS‐related symptoms as comorbidity [20, 21].

### 2.6 Statistical Analyses

Cohort characteristics were described using counts with proportions for categorical variables and means with standard deviations (SD) for continuous variables. We reported the hazard ratio (HR) with a 95% confidence interval (CI) from the Cox proportional hazard model. We used SAS 9.4 for analytic dataset preparation and R 4.2.1 for all statistical analyses.

#### 2.6.1 Analysis With Investigator‐Specified Covariates

We used the Kaplan–Meier curve to show the survival probability of people with DMD exposure versus no DMD exposure. To explore the adjusted relationship between exposure to any DMD and time to all‐cause mortality, we fitted the Cox proportional hazards model, adjusting for investigator‐specified covariates. Here, the investigator‐specified covariates were age, sex, neighborhood income quintile, comorbidity status, and calendar year [5].

#### 2.6.2 Analysis With Additional High‐Dimensional Proxy Data

We implemented three versions of high‐dimensional propensity score (hdPS) and three versions of high‐dimensional disease risk score (hdDRS) methods [7, 23] that incorporated these proxy information as empirical covariates (binary proxies). Briefly, there were seven steps in hdPS and hdDRS analyses. First, proxy information was obtained from three linked data sources with a one‐year covariate assessment period prior to the index date: (i) physician visits: 3‐digit ICD‐9 diagnostic codes, (ii) hospitalizations: 3‐digit ICD‐9/10 diagnosis codes and procedure codes, and (iii) prescriptions dispensed: drug identification numbers. Second, ICD‐9/10 codes used to define the Charlson comorbidity index and MS, as well as MS‐specific drug identification numbers, were excluded to avoid double counting [8]. Third, recurrence of proxies/codes was converted into binary variables, resulting in 1403 empirical covariates. Fourth, the Bross formula was used in hdPS‐1, hdPS‐2, hdDRS‐1, and hdDRS‐2, while LASSO regression was applied in HdPS‐3 and hdDRS‐3. Fifth, the top 200 covariates prioritized by bias were selected in hdPS‐1, hdPS‐2, hdDRS‐1, and hdDRS‐2. In HdPS‐3 and hdDRS‐3, all covariates with nonzero coefficients were included. Sixth, logistic regression (hdPS‐1, hdDRS‐1) or LASSO (hdPS‐2, hmethodsdPS‐3, hdDRS‐2, hdDRS‐3) was used to estimate scores. Seventh, predicted scores were categorized into deciles and incorporated into the Cox proportional hazards model alongside investigator‐specified covariates. See Box S1 for additional details of the steps.

## 3 Results

### 3.1 Cohort Characteristics

We identified 19 360 people with MS, with 214 332 person‐years of follow‐up (Table S2). The mean (SD) age at index was 44.5 (13.5) years, 72.0% were females, and 22.3% had at least one comorbidity. Among these participants, 17.0% (*n* = 3287) were exposed to any DMD in the first 5 years. People with any versus no DMD exposure were comparatively younger at the index date (mean age 37.5 vs. 45.9 years) and more likely to have a comorbidity (15.9% vs. 23.5%).

#### 3.1.1 Results From the Analysis With Investigator‐Specified Covariates

**FIGURE 1:**
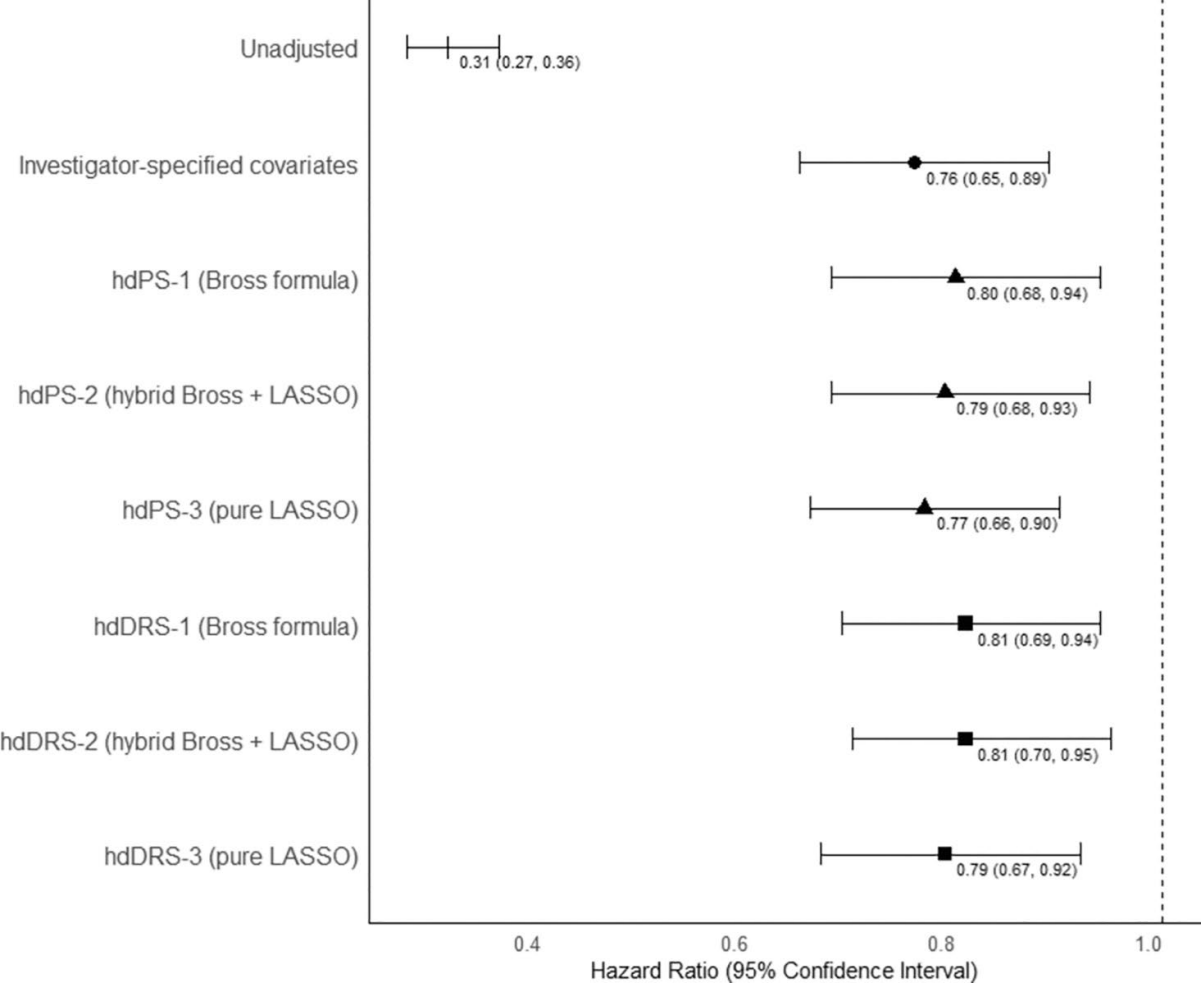
Forest plot of hazard ratios obtained from various methods under consideration depicting the relationship between exposure to any disease‐modifying drug (DMD) for multiple sclerosis and all‐cause mortality in British Columbia, Canada, 1996–2017.

Figure S1 shows the Kaplan–Meier curve, where people with any DMD exposure had a significantly higher survival probability than those without DMD exposure. In the unadjusted analysis, we observed a 69% lower risk of mortality associated with any DMD exposure (HR 0.31; 95% CI: 0.27–0.36) (Figure 1, Table S3). Our adjusted analysis found a 24% lower risk of mortality associated with any DMD exposure (adjusted HR [aHR]: 0.76, 95% CI: 0.65–0.89).

In sensitivity analyses, after repeating the analyses with any DMD exposure at 3, 4, and 6 years, the aHRs (95% CI) were 0.75 (0.62–0.91), 0.76 (0.65–0.90), and 0.73 (0.63–0.84), respectively, after adjusting for the same investigator‐specified covariates. A previous study that considered DMD as a time‐varying exposure and adjusted for similar investigator‐specified covariates estimated an aHR of 0.74 (95% CI: 0.56–0.98) [5].

#### 3.1.2 Results From Analysis With Additional High‐Dimensional Data

In the hdPS analyses, we observed a 20%–23% lower mortality risk among people with any DMD exposure than no DMD exposure (hdPS‐1 aHR: 0.80, 95% CI: 0.68–0.94; hdPS‐2 aHR: 0.79, 95% CI: 0.68–0.93; hdPS‐3 aHR: 0.77, 95% CI: 0.66–0.90) (Figure 1, Table S3). The hdDRS analyses also revealed a 19%–21% lower mortality risk associated with any DMD exposure (hdDRS‐1 aHR: 0.81, 95% CI: 0.69–0.94; hdDRS‐2 aHR: 0.81, 95% CI: 0.70–0.95; hdDRS‐3 aHR: 0.79, 95% CI: 0.67–0.92). Sensitivity analyses for hdPS with inverse probability weighting (see Figure S2 for an SMD plot), analysis with the top 500 or all empirical covariates resulted in a 16%–33% lower mortality risk associated with any DMD exposure (Table S4). Table S5 summarizes the mitigation strategies we applied to address the challenges associated with these analytical methods [8].

## 4 Conclusions

In this brief report, we aimed to incorporate high‐dimensional proxy information to reduce potential residual confounding. After including proxy information from three additional data dimensions using hdPS and hdDRS frameworks, we found that the results from hdPS and hdDRS methods were not materially different from those obtained using investigator‐specified covariates. This suggests that, at least in the current application, the added advantage of using high‐dimensional proxy data could not be demonstrated.

We acknowledge that while the “never vs. ever” exposure definition was chosen for its intuitive interpretation of treatment effect, this approach may introduce challenges related to prevalent user bias. Although we opted to avoid a time‐varying analysis in favor of simplicity, adopting the “never vs. ever” definition within a 5‐year period may still affect the interpretation of our results. Our sensitivity analyses, which considered alternative exposure periods of 3, 4, and 6 years, as well as the consistency of our findings with a previous study that considered time‐varying exposure (although technically targeting different estimands) [5], suggest that our choice of the 5‐year exposure period may not significantly impact the overall conclusions of this case study.

The hdPS and hdDRS methods offer a structured approach to addressing residual confounding, their effectiveness will be limited by the data at hand [7, 23]. In this study, we used the most recent of the first MS or related demyelinating diagnostic code, or MS DMD prescription filled to determine the index date, aligning the start of follow‐up with a clinically relevant event, such as first clinical evidence of demyelination (recorded as an ICD code) or treatment initiation [5, 24]. While this approach ensures the index date reflects an important point in the patient's clinical trajectory, it may introduce methodological challenges, particularly the potential for immortal time bias [25].

Nevertheless, we find it reassuring that the results from the investigator‐specified analysis align in magnitude and direction with those incorporating high‐dimensional data proxies. This convergence suggests that, in this instance, traditional confounder adjustment was likely sufficient. However, this should not detract from the potential value of hdPS and hdDRS methods in other contexts, where more relevant and robust proxy data might be available. Given the limitations acknowledged above, future research should aim to incorporate more diverse and relevant data dimensions and attempt to replicate these findings in other datasets.

## Author Contributions

Mohammad Ehsanul Karim conceptualized the study, supervised the project, led funding acquisition and resources, led the formal analysis, original drafting of the manuscript and contributed equally to the review and editing of the manuscript. Md. Belal Hossain conducted the formal analysis, software development, updating of the manuscript, and contributed equally to the investigation, methodology, and review. Huah Shin Ng contributed to the validation of the results, provided supporting funding, and contributed equally to the review and editing of the manuscript. Feng Zhu supported funding acquisition, contributed to the validation of the findings, and equally participated in the review and editing of the manuscript. Hanna A. Frank contributed to the validation of the findings, and equally participated in the review and editing of the manuscript. Helen Tremlett shared responsibilities for data curation and resources, provided supporting funding, and contributed equally to the review and editing of the manuscript.

## Ethics Statement

The studies involving human participants were reviewed and approved by the University of British Columbia Clinical Research Ethics Board and British Columbia Ministry of Health.

## Consent

Written informed consent to participate in this study was provided by the participants' legal guardian/next of kin.

## Conflicts of Interest

M.E.K.'s research is supported by MEK's Natural Sciences and Engineering Research Council of Canada (NSERC) Discovery Grants and Discovery Accelerator Supplements. M.E.K. was also supported by the Michael Smith Foundation for Health Research Scholar award. Over the past 4 years, author M.E.K. has received consulting fees from Biogen (unrelated to the current project) and participated in Advisory Boards and/or Satellite Symposia of Biogen Inc. H.S.N. is supported by a Beat Cancer Early Career Research Fellowship from Cancer Council South Australia and has also received funding from the Southern Adelaide Local Health Network Enquiry Grant. H.T. has, in the last 5 years, received research support from the Canada Research Chair Program, the National Multiple Sclerosis Society, the Canadian Institutes of Health Research, Multiple Sclerosis Canada, the Multiple Sclerosis Scientific Research Foundation and the EDMUS Foundation (‘Fondation EDMUS contre la sclérose en plaques’). In addition, in the last 5 years, has had travel expenses or registration fees prepaid or reimbursed to present at CME conferences or attend meetings (as a member of the International Advisory Committee on Clinical Trials in Multiple Sclerosis) from the Consortium of MS Centres (2023), the Canadian Neurological Sciences Federation (2023), National MS Society (2022, 2023, 2024), ECTRIMS/ACTRIMS (2017–2024), American Academy of Neurology (2019). Speaker honoraria are either declined or donated to an MS charity or to an unrestricted grant for use by HT's research group. The other authors declare no conflicts of interest.

## Supporting information


**Appendix S1.** Supporting Information.

## Data Availability

Access to data provided by the Data Stewards is subject to approval but can be requested for research projects through the Data Stewards or their designated service providers. The following data sets were used in this study: Consolidation files, Hospital Separations, Medical Services Plan (MSP), PharmaNet, Vital Events and Statistics—Deaths. You can find further information regarding these data sets by visiting the PopData project webpage at: https://my.popdata.bc.ca/project_listings/18‐120/collection_approval_dates. All inferences, opinions, and conclusions drawn in this publication are those of the authors and do not reflect the opinions or policies of the Data Stewards.
