# Supplementary material for: Evaluating the Role of High‐Dimensional Proxy Data in Confounding Adjustment in Multiple Sclerosis Research: A Case Study
Source: Pharmacoepidemiol Drug Saf. 2025 Feb 3;34(2):e70112. doi: 10.1002/pds.70112 (PMC11791124; doi:10.1002/pds.70112)
Supplement: Supplementary file 1 — Appendix S1. Supporting Information. [file PDS-34-e70112-s001.pdf]

Appendix eTable1: The drug identification numbers for multiple sclerosis specific disease-modifying drugs.

Disease-modifying drug	***Drug identification number***
Beta-interferon	02169649, 02337819, 02444399, 02444380, 02444372, 02444402, 02237770, 02269201, 02281708, 02277492, 02237317, 02237319, 02237320, 02318253, 02318261, 02318288
Glatiramer acetate	02233014, 02245619, 02441446, 02456915, 02460661
Natalizumab	02286386
Fingolimod	02365480
Dimethyl fumarate	02404508, 02420201
Teriflunomide	02416328
Alemtuzumab	02418320
Daclizumab	02459620, 02459639
Ocrelizumab	02467224

Appendix eTable 2: Characteristics of the study participants with any disease-modifying drug (DMD) for multiple sclerosis versus no DMD in British Columbia, Canada, 1996-2017, stratified by DMD exposure status.

CHARACTERISTICS	TOTAL (N = 19,360)	NO DMD (N = 16,073)	ANY DMD (N = 3,287)	SMD
**FOLLOW-UP TIME (YEARS)**	214,332	178,387	35,945	
**AGE IN YEARS, MEAN (SD)**	44.52 (13.54)	45.94 (13.74)	37.54 (9.93)	0.701
**SEX**				0.004
Female	13940 (72.00)	11568 (71.97)	2372 (72.16)	
Male	5420 (28.00)	4505 (28.03)	915 (27.84)	
**NEIGHBORHOOD INCOME QUINTILE**				0.038
Lowest 20%	3763 (19.44)	3143 (19.55)	620 (18.86)	
Lower 20%	3695 (19.09)	3092 (19.24)	603 (18.34)	
Middle 20%	4029 (20.81)	3338 (20.77)	691 (21.02)	
Higher 20%	4094 (21.15)	3395 (21.12)	699 (21.27)	
Highest 20%	3779 (19.52)	3105 (19.32)	674 (20.51)	
**CHARLSON COMORBIDITY SCORE**				0.216
0	15051 (77.74)	12288 (76.45)	2763 (84.06)	
1	2979 (15.39)	2583 (16.07)	396 (12.05)	
2	855 (4.42)	755 (4.70)	100 (3.04)	
≥3	475 (2.45)	447 (2.78)	28 (0.85)	
**CALENDAR YEAR**				0.497
1996-1999	8533 (44.08)	7710 (47.97)	823 (25.04)	
2000-2005	3905 (20.17)	3051 (18.98)	854 (25.98)	
2006-2011	3722 (19.23)	2926 (18.20)	796 (24.22)	
2012-2017	3200 (16.53)	2386 (14.84)	814 (24.76)	

Abbreviation *– DMD: disease-modifying drug (DMD), SD: standard deviation, SMD: standardized mean difference.*

Appendix eTable 3: Relationship between exposure to any disease-modifying drug (DMD) for multiple sclerosis and all-cause mortality in British Columbia, Canada, 1996-2017.

Analysis	*HR*	*95% CI*
Unadjusted	0.31	0.27-0.36
Adjusted investigator-specified covariates [a]	0.76	0.65-0.89
hdPS-1 (original algorithm with proxies selected via Bross formula) [1]	0.80	0.68-0.94
hdPS-2 (hybrid LASSO-based hdPS) [2]	0.79	0.68-0.93
hdPS-3 (pure LASSO-based hdPS) [3]	0.77	0.66-0.90
hdDRS-1 (hdDRS with proxies selected via Bross formula) [1]	0.81	0.69-0.94
hdDRS-2 (hybrid LASSO-based hdDRS) [2]	0.81	0.70-0.95
hdDRS-3 (pure LASSO-based hdDRS) [3]	0.79	0.67-0.92

Abbreviation *– CI: confidence interval; HR: hazard ratio; hdPS: high-dimensional propensity score; hdDRS: high-dimensional disease risk score.*

[a] Adjusted the model for age, sex, neighborhood income quintile, comorbidity status, and calendar year. The E-value was calculated based on a non-rare outcome, with an observed outcome frequency of 16.34%. Given the E-value of 1.71 for the point estimate for this analysis, an unmeasured confounder would need to be associated with both DMD use and mortality by a hazard ratio of at least 1.71 to fully explain away the observed effect, which seems unlikely based on our understanding from the literature. The E-value of 1.39 for the lower bound of the confidence interval indicates that even a moderately strong unmeasured confounder would be insufficient to reduce the association to the null, further supporting the robustness of our results. The E-value is typically used when we suspect there may be unmeasured confounders that have not been accounted for. Since the hdPS/hdDRS methods are aimed at addressing this by adding additional proxy variables, we did not calculate E-values for those methods, as these hdPS/hdDRS methods themselves already serve as a form of sensitivity analysis.

[1] The Bross formula was used to prioritize empirical covariates, and logistic regression was fitted to estimate the propensity scores or disease risk scores.

[2] The Bross formula was used to prioritize empirical covariates. The LASSO model was fitted to further refine the empirical covariate list, and use those refined empirical covariate list to estimate the propensity scores or disease risk scores.

[3] The LASSO model was used to find empirical covariates, and another LASSO model was fitted to estimate the propensity scores or disease risk scores.

Appendix eTable 4: Sensitivity analyses in exploring the relationship between exposure to any disease-modifying drug (DMD) for multiple sclerosis and all-cause mortality in British Columbia, Canada, 1996-2017.

Analysis	*HR*	*95% CI*
hdPS with inverse probability weighting [a]		
hdPS-1 (original algorithm with proxies selected via Bross formula) [1]	0.68	0.55-0.83
hdPS-2 (hybrid LASSO-based hdPS) [2]	0.67	0.55-0.82
hdPS-3 (pure LASSO-based hdPS) [3]	0.70	0.58-0.84
Analyses with 500 empirical covariates [b]		
hdPS-1 (original algorithm with proxies selected via Bross formula) [1]	0.81	0.69-0.94
hdPS-2 (hybrid LASSO-based hdPS) [2]	0.79	0.68-0.93
hdPS-3 (pure LASSO-based hdPS) [3]	0.76	0.65-0.89
hdDRS-1 (hdDRS with proxies selected via Bross formula) [1]	0.81	0.69-0.94
hdDRS-2 (hybrid LASSO-based hdDRS) [2]	0.82	0.70-0.96
hdDRS-3 (pure LASSO-based hdDRS) [3]	0.82	0.70-0.96
Analyses with all 2,546 empirical covariates [c]		
hdPS with logistic [4]	0.81	0.69-0.96
hdPS LASSO [5]	0.75	0.64-0.87
hdDRS with logistic [4]	0.67	0.57-0.78
hdDRS with LASSO [5]	0.84	0.72-0.98

Abbreviation – *CI: confidence interval; HR: hazard ratio; hdPS: high-dimensional propensity score; hdDRS: high-dimensional disease risk score.*

[a] *Stabilized inverse probability of being DMD exposed was estimated. The mean weight was 0.99 for all three hdPS methods with a minimum of 0.21, 0.23, and 0.024, and a maximum of 11.93, 8.80, and 8.48. Age was imbalanced in terms of standardized mean difference for all methods and thus adjusted for in the outcome model.*

[b] *Investigator-specified and the top 500 empirical covariates were used.*

[c] *Investigator-specified and all empirical covariates without prioritizing them were used.*

[1] *The Bross formula was used to prioritize empirical covariates, and logistic regression was fitted to estimate the propensity scores or disease risk scores.*

[2] *The Bross formula was used to prioritize empirical covariates. The LASSO model was fitted to further refine the empirical covariate list, and use those refined empirical covariate list to estimate the propensity scores or disease risk scores.*

[3] *The LASSO model was used to find empirical covariates, and another LASSO model was fitted to estimate the propensity scores or disease risk scores.*

[4] *Logistic regression was fitted to estimate the propensity scores or disease risk scores.*

[5] *LASSO was fitted to estimate the propensity scores or disease risk scores.*

Appendix eTable 5: Challenges and Mitigation Strategies in High-Dimensional Propensity Score (hdPS) and Disease Risk Score (hdDRS) Modeling

Challenge	Description	Potential Solution
Model Performance	hdPS/hdDRS models are data-driven, potentially including irrelevant variables, which may affect performance by inflating variance.	Regularly check balance diagnostics (e.g., standardized mean differences) to ensure adequate covariate balance and follow best practices for fitting propensity score models, and conduct the below-mentioned sensitivity analyses for hdPS/hdDRS models to understand how the inclusion of proxy variables have impacted the results.
Overfitting and Proxy Selection Uncertainty	Including too many irrelevant or correlated proxies may result in overfitting and inflated variance.	Conduct sensitivity analyses by varying the number of selected proxies (k) and assess the stability of effect estimates to ensure robustness.
Multicollinearity	Correlation between hdPS variables may lead to multicollinearity, increasing standard errors and reducing model reliability.	Use machine learning methods such as LASSO or hybrid-approaches to automatically select the most relevant variables and reduce collinearity issues.
Instrumental Variables	The inclusion of variables that act as instruments (affecting exposure but not outcome) can introduce bias.	Manually review variables and apply clinical judgment to exclude potential instruments and problematic proxies. Unfortunately, there is no straightforward data-driven solution for this issue.
Unmeasured Confounding	It is important to acknowledge that the effectiveness of hdPS or hdDRS methods hinges on the assumption that the selected empirical covariates can collectively serve as proxies for all unmeasured or residual confounders. This is a strong assumption and one that is empirically unverifiable. The extent to which these methods can reduce or eliminate residual confounding is likely contingent upon the availability of a database rich in relevant and correlated proxy information.	Acknowledge this limitation in the interpretation of results, and ensure that sensitivity analyses (e.g., E-values) are used to assess the potential impact of unmeasured confounders on the findings.

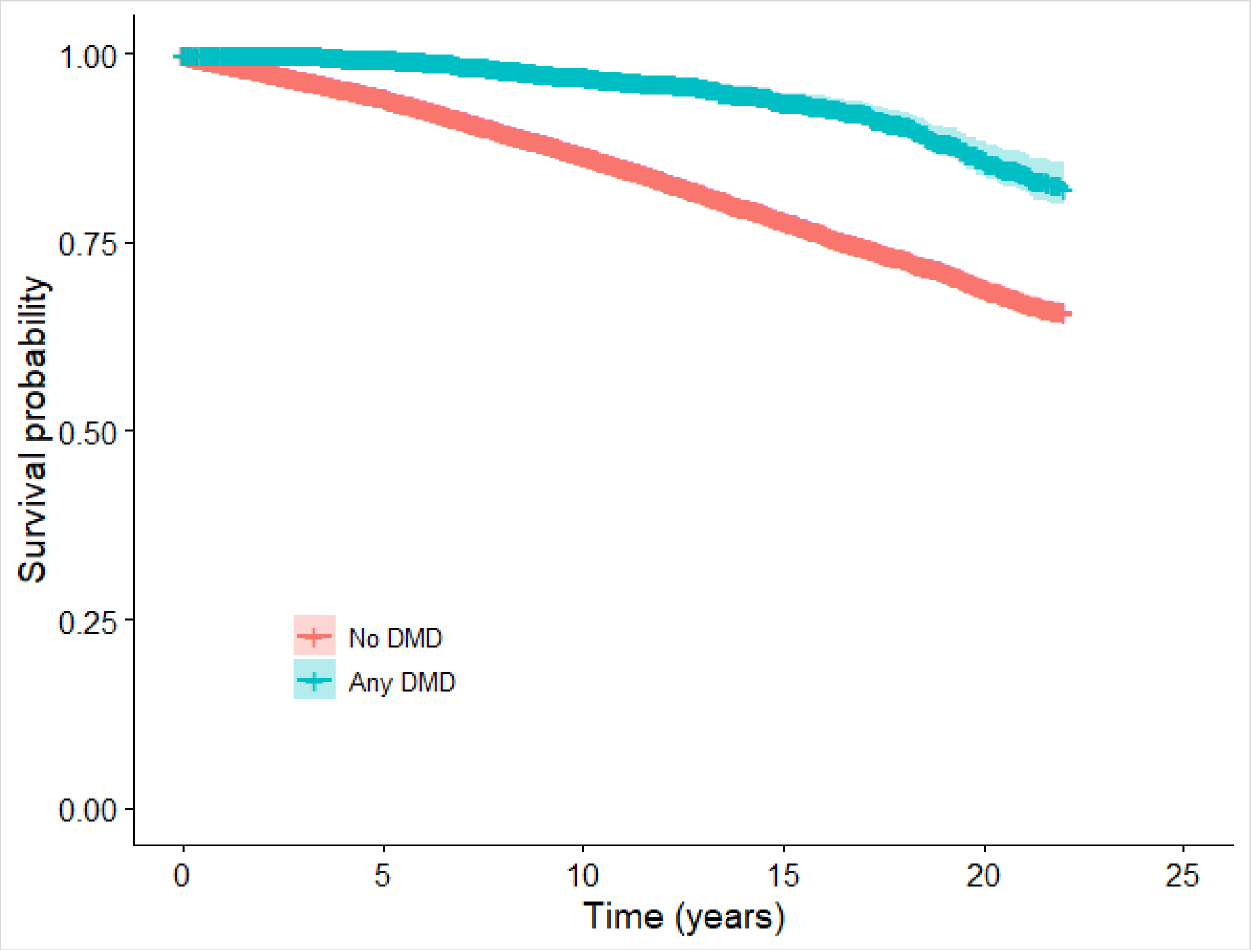

Number-at risk	*Time (years)*					
	0	5	10	15	20	25
No DMD	16,073	12,158	8,025	5,363	3,202	*
Any DMD	3,287	2,451	1,702	1,036	438	*

Appendix eFigure 1: Kaplan-Meier plot for all-cause mortality risk among people with any disease-modifying drug (DMD) for multiple sclerosis versus no DMD in British Columbia, Canada, 1996-2017. Notes: an asterisk (*) indicates suppressed due to cell count <5, or cross-suppressed as the next lowest value.

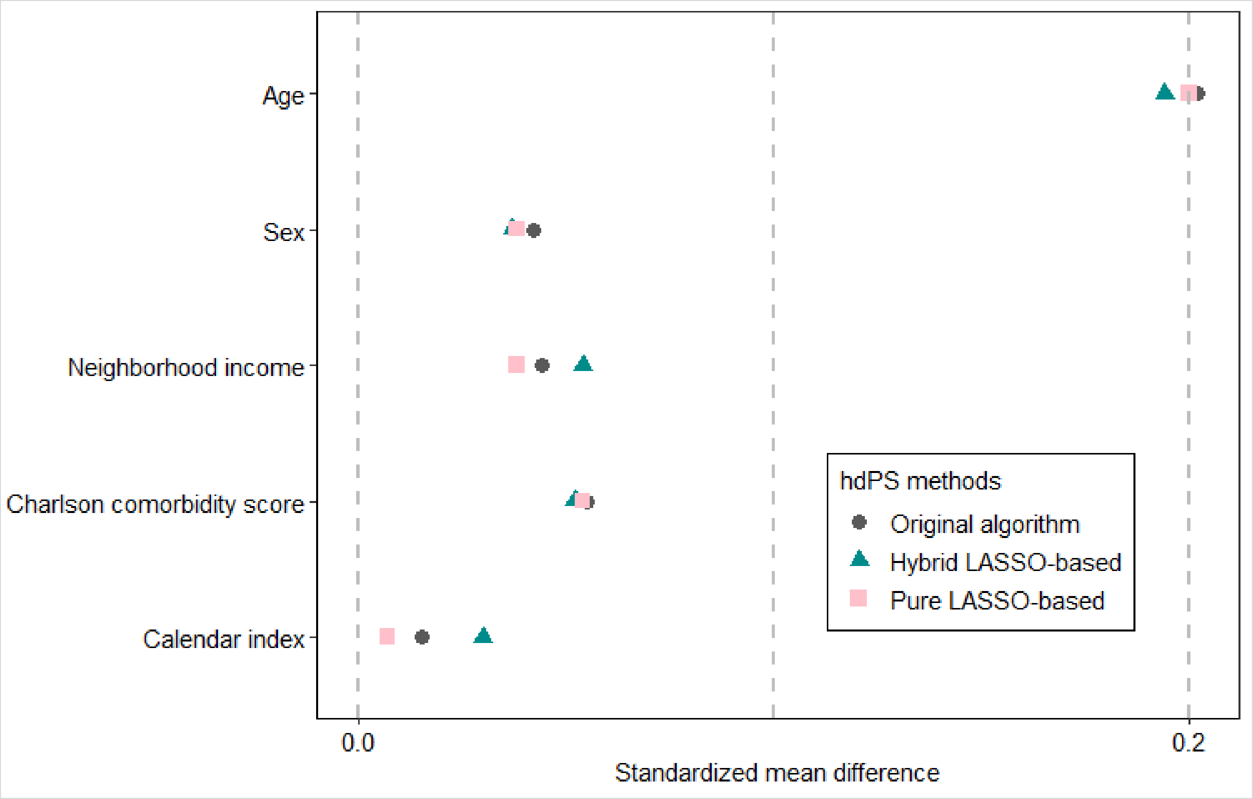

Appendix eFigure 2: Standardized mean difference for the high-dimensional propensity score (hdPS) methods with inverse probability of stabilized weighting. Original algorithm: The Bross formula was used to prioritize empirical covariates, and logistic regression was fitted to estimate the propensity scores or disease risk scores; Hybrid LASSO-based: The Bross formula was used to prioritize empirical covariates. The LASSO model was fitted to further refine the empirical covariate list, and use those refined empirical covariate list to estimate the propensity scores or disease risk scores; Pure LASSO-based: The LASSO model was used to find empirical covariates, and another LASSO model was fitted to estimate the propensity scores or disease risk scores.

Appendix eBox 1: List of algorithms used to incorporate high-dimensional proxy information using the high-dimensional propensity score and disease risk score frameworks.

hdPS-1 (original algorithm with proxies selected via Bross formula): There were seven steps of hdPS:

- **Step 1** – identify the source of empirical or proxy covariates: Similar to the investigator-specified covariates, all empirical covariates were identified in a one-year covariate assessment window prior to the cohort entry date.
- **Step 2** – Empirical covariates identification: All codes in each data dimension were considered, resulting in 1,403 codes/proxies.
- **Step 3** – Assessing recurrence of codes: We generated three binary recurrence covariates for each of the candidate empirical covariates: (i) once, (ii) frequent, and (iii) sporadic. There was a total of 2,546 empirical covariates.
- **Step 4** – Prioritizing empirical covariates: We used the Bross formula[1] to prioritize the covariates.
- **Step 5** – Empirical covariate selection: We selected the top 200 empirical covariates based on the log of bias calculated in Step 4.
- **Step 6** – predicting propensity scores: We estimated the propensity scores by fitting logistic regression, with investigator-specified variables (see 'Analysis with investigator-specified covariates') and empirical covariates from Step 5.
- **Step 7** – outcome modelling: The outcome model was the Cox proportional hazards model, adjusting for the deciles of propensity scores and investigator-specified covariates.[2] A robust sandwich-type variance estimator was used to estimate the 95% CI.

hdPS-2 (hybrid LASSO-based hdPS): HdPS-2 is similar to hdPS-1, except for Step 6, where we estimated the propensity scores by fitting LASSO regression to deal with the overfitting of the propensity score model. We forced the model to keep all investigator-specified covariates but variable selection only for empirical covariates. Hyperparameters of the model were chosen using 5-fold cross-validation.

hdPS-3 (pure LASSO-based hdPS): HdPS-3 is similar to hdPS-2, except for Steps 4 and 5:

- **Step 4** – prioritizing empirical covariates: We used the LASSO method to prioritize the empirical covariates, without using the Bross formula.[2] The 5-fold cross-validation was used to choose the hyperparameters of the model.
- **Step 5** – Empirical covariate selection: We selected all empirical covariates selected based on LASSO model in Step 4.

hdDRS-1 (hdDRS with proxies selected via Bross formula): Like hdPS, there were also seven steps of hdDRS. Steps 1-5 for hdDRS-1 are identical to Steps 1-5 for hdPS-1. Steps 6 and 7 for hdDRS-1 are as follows:

- **Step 6** – predicting disease risk scores: In this step, we fitted the outcome model with investigator-specified variables and empirical covariates from step 5 on the cohort of unexposed to any DMD. Then we fitted logistic regression (binary all-cause mortality as the outcome variable) to estimate the disease risk score.[3]
- **Step 7** – outcome modelling: The outcome model was the Cox proportional hazards model, adjusting for the deciles of disease risk scores and investigator-specified covariates. We used a robust sandwich-type variance estimator to estimate the 95% CI.

hdDRS-2 (hybrid LASSO-based hdDRS): hdDRS-2 is similar to hdDRS-1, except for Step 6, where we estimated the disease risk scores by fitting LASSO regression to deal with the overfitting of the model.[3] Again, we forced the model to keep all investigator-specified covariates but do the variable selection only for empirical covariates. Hyperparameters of the model were chosen using 5-fold cross-validation.

hdDRS-3 (pure LASSO-based hdDRS): hdDRS-2 is analogous to hdPS-3, with the LASSO model for empirical covariate selection and another LASSO model for estimating disease risk scores.

eReferences

1. Schneeweiss, S., Rassen, J. A., Glynn, R. J., Avorn, J., Mogun, H. & Brookhart, M. A. High-dimensional propensity score adjustment in studies of treatment effects using health care claims data. *Epidemiology* **20,** 512 (2009). doi:10.1097/EDE.0b013e3181a663cc
2. Karim, M. E., Pang, M. & Platt, R. W. Can we train machine learning methods to outperform the high-dimensional propensity score algorithm? *Epidemiology* **29,** 191–198 (2018). doi:10.1097/EDE.0000000000000787
3. Kumamaru, H., Schneeweiss, S., Glynn, R. J., Setoguchi, S. & Gagne, J. J. Dimension reduction and shrinkage methods for high dimensional disease risk scores in historical data. *Emerging themes in epidemiology* **13,** 1–10 (2016).
